# Emotional and external eating styles associated with obesity

**DOI:** 10.1186/s40337-023-00797-w

**Published:** 2023-05-02

**Authors:** Hassiba Benbaibeche, Hamza Saidi, Abdenour Bounihi, Elhadj Ahmed Koceir

**Affiliations:** 1 Faculté des sciences, département des sciences de la nature et de la vie, Université Alger 1, Alger, Algérie; 2 University of Sciences and Technology Houari Boumediene, Algiers, Algeria; 3 Ecole Supérieure des Sciences de l’Aliment et des Industries Agroalimentaires, Alger, Algérie

**Keywords:** Obesity, Pathological eating, Emotional eating, External eating, DEBQ

## Abstract

**Background:**

Obesity is related to eating habits. Overeating is the most behavioural trait implicated in obesity; emotional, external and rigid restrained eating are three maladaptive eating habits that are associated to overeating.

**Objectives:**

The current study assesses the eating styles of Algerian adults. It identifies and analyses differences in eating styles in a sample from adults with normal BMI and who have obesity. The study examines the relationship between eating styles and BMI.

**Methods:**

The sample consisted of 200 volunteers aged from 31 to 62 years old, 110 with obesity and 90 having normal BMI. The participants were recruited from hospital and university employees. They were questioned about their eating habits. The participants did not receive any treatment. To assess eating styles, participants completed the DEBQ.

**Results:**

The prevalence of women was in the majority, representing 61% (n = 122) in the total sample (63.63% (n = 70) with obesity, and 55.77% (n = 52) with normal BMI). The prevalence of men represents 39% (n = 78) in the total sample (36.36% (n = 40) with obesity, and 42.22% (n = 38) with normal BMI). Participants with obesity showed pathological eating styles. They scored higher on emotional and external eating styles than to normal BMI group. However, restraint eating showed a slight no significant increase. The mean scores ± standard deviations observed in each eating styles were: emotional eating (2.88 ± 0.99** vs. 1.71 ± 0.32), external eating (3.31 ± 0.68** vs. 1.96 ± 0.29), and retrained eating (1.81 ± 0.7^ns^ vs. 1.3 ± 0.30). The linear regression analysis showed an effect of emotional and external eating on BMI.

**Conclusion:**

These results could be used to provide clinical information at the initial screening for obesity criteria, obesity prevention and treatment.

## Abstract (summary)

Emotional, external and rigid restrained eating are three eating habits related to obesity. They are associated to overeating in response to negative emotions, external food-related cues, and body weight control. Obesity treatment necessarily requires the training of medical professionals. The objective of this research is to assess eating styles of people living with obesity and to analyse differences comparing with people with normal body mass index (BMI). We examined the relationship between eating habits and BMI. A total of 200 participants aged from 31 to 62 years old were recruited from hospital and university workers; 110 with obesity and 90 with normal BMI. The Dutch Eating Behaviour Questionnaire was used to assess eating styles (DEBQ). The outcomes of the current study showed that people with obesity exhibit a high emotional and external eating. However, they show a slight restraint eating. BMI was associated to both emotional and external eating. Negatives emotions lead participants to overeat as a response way to cope with, and expose them to obesity. These results are important for the initial screening of obesity criteria. For prevention and treatment of obesity, eating styles must be targeted as factors associated to obesity in order to cope with negative emotions.

## Background

The prevalence of obesity has increased worldwide and has reached pandemic proportions in the present century [1]. Obesity is associated with the development of important non-communicable chronic diseases, involving cardiovascular diseases, hypertension, type 2 diabetes mellitus and hyperlipidemia [2].

More than two billion people worldwide are with overweight or obesity, 1.9 billion individuals are considered to be overweight and 650 million adults are living with obesity in 2016, representing 39% and 19% respectively, of world population [3]. In Algeria, the number of individuals with obesity cases is largely increasing, where 16.6% men and 28.7% females were affected by obesity in 2014 [1]. In 2018, based on the WHO STEPwise approach, according to the latest national survey on the measurement of risk factors for non-communicable diseases, the prevalence of obesity and overweight in Algeria exceeds 50% of the population [4].

Beside the interaction between genetic predispositions, sedentary lifestyle and our living environment [5], eating habits play a key role in the pathogenesis of obesity. Pathological eating habits are related to a substantial proportion of deaths from heart disease, stroke, and type 2 diabetes [6].

Currently, over the past four decades, eating habits changed significantly through a great consumption of processed food that are high in energy and with a high glycaemic index leading people to overeat [7]. In the modern food environment, availability of palatable food, high in sugar and fat, surpass the rewarding properties of non-processed food [8].

In Algeria, nutritional transition deviate eating habits from the traditional Mediterranean diet to modern fast food style [9] and towards high consumption of fatty and sweet food [10]. The Mediterranean diet has been associated with a lower risk of obesity and type 2 diabetes mellitus [11].

Overeating is the most behavioural trait implicated in obesity; emotional, external and restrained eating are three maladaptive eating habits that lead to overeating. Emotional eating is the tendency to overeat in response to arousal states such as anger, fear, and anxiety [12]; it is associated to the intake of sweet and energy-dense food [13, 14], binge eating [15] and is considered as a risk factor for obesity [16].

External eating describes eating in response to external food-related cues (e.g., sight and smell of food), regardless of internal signals of hunger and satiety [17]. It is involved in increasing prevalence of obesity and predicted food cravings [18, 19]. The availability of high dietary variety and palatable food in the modern food environment [20, 21] can be considered as cues for overeating.

Restrained eating involves the intention to restrict food intake in order to control body weight contrary to physiological control, such as hunger and satiety [22]. Food restraint is a cognitive causal factor in weight gain [23]. However, it was associated with reduced food intake [24]. While rigid restraint has been associated with a tendency to overeat and poor weight control, flexible restraint predicts better weight control and reduced overeating [25]. The current study aims to elucidate three main objectives. The first one is to assess eating styles of Algerian adults as measured by the DEBQ. We conducted the study in Algerian population as a contribution to the literature of previously-published work using the DEBQ in others populations. In the current study, we validated the DEBQ in Algerian population of participants. The second is to identify and analyse differences in eating styles in a sample from adults with normal BMI and who have obesity. Finally, the third objective is to examine the relationship between eating styles and BMI. We expected that people with obesity would have pathological eating style when compared to normal BMI individuals. We hypothesised that pathological eating styles were related to high BMI.

## Methods

### Participants

Two hundred Algerian volunteers (107 women and 93 men) were recruited to participate in this anonymous study of eating styles. The total sample consisted of 110 with obesity and 90 with normal BMI participants aged from 31 to 62 years old. A self-administered questionnaire was conducted in 2021 at the Mustapha Pacha University hospital. The participants were recruited from hospital and university workers. From the 220 people invited, 200 responded positively and accepted to participate in the current study. The participants did not receive any treatment and were interrogated for any eating habits. Exclusion criteria were diabetes, smoking habits, cardiovascular diseases and medications that could affect feeding behaviour. The study was conducted according to the guidelines of the Declaration of Helsinki. All participants provided written informed consent.

### Measures

#### Anthropometry

We measured height, body weight, and waist circumference (WC) of the participants using a standardised protocol. The BMI was calculated by dividing weight in kilograms by the square of the height in metres (kg/m^2^). The BMI variable was divided into 2 categories: normal (18.5–24.9 kg/m^2^) and high (≥ 30 kg/m^2^). Body fat per cent (BF %) was also calculated for each participant according to the formula [26]:

$${\text{BF}}\% = 1.2 \times {\text{BMI}} + 0.23 \times {\text{age}} - 10.8 \times {\text{sex}} - 5.4 \;{\text{sex}}({\text{males}} = 1,{\text{females}} = 0).$$

The BMI is widely used to measure obesity in people. However, other ways are used as the WC and the BF% [27]. BMI cannot assess the BF% [28] and WC is a better predictor over BMI at determining BF% in male participants [29]. People with a high BMI and high proportion of fat-free mass can be wrongly categorised with overweight or obesity [30]. In the present study, we used the three measures; BMI, WC and BF% to increase validity of obesity description. We measured the WC and the BF% to evaluate abdominal and total adiposity respectively.

#### Eating styles assessment

The DEBQ was used to assess eating behaviour [12]. The DEBQ is one of the most frequently used self-report measures to assess three eating styles: emotional eating (13 items), external eating (10 items) and restrained eating (10 items). The DEBQ focuses on eating in response to negative emotions. Participants respond to 33 items using a scale ranging from never [1] to very often [5]. The final score on each scale is the average of the item scores on that scale. In the current study, we included a reliable sample size, in reference to eating disorder statistics worldwide that estimated the prevalence at 7.8% [31]. We also validated the DEBQ in Algerian participants with obesity and normal BMI. The French version of DEBQ showed a good reliability. The Cronbach’s alpha was 0.9 for emotional eating, 0.87 for external eating, and 0.83 for restrained eating.

### Statistical analysis

Statistical analysis was carried out with SPSS, version 25 (SPSS, Inc., Chicago, IL, USA). Quantitative data such as age, BMI and BF% were analysed with the Student t-test. Categorical variables, such as eating behaviour were compared with Chi-squared test. The results were presented as mean and standard deviations. A univariate logistic regression was used to evaluate associations between the DEBQ score and BMI. The level of statistical significance for the evaluation of the results was *p* < 0.05.

## Results

Participant characteristics are summarised in Table 1 according to their BMI category and also in the total sample. The prevalence of women was in the majority representing 61% (n = 122) in the total sample (63.63% (n = 70) with obesity, and 55.77% (n = 52) with normal BMI). The prevalence of men represents 39% (n = 78) in the total sample (36.36% (n = 40) with obesity, and 42.22% (n = 38) with normal BMI). An increase was observed in WC, BMI and BF% across the individuals with obesity in comparison with normal BMI group which is in favour of abdominal and total adiposity.

**Table 1:** Characteristics of participants according to BMI

Variables		High BMI (n = 110)	Normal BMI (n = 90)		All participants (n = 200)
Gender (female)		63.63% (70)*	55.77% (52)		61% (122)
Age (years)		41.73 (9.69)*	35 (8.62)		38.31 (11.35)
BMI (Kg/m^2^)		33.31 (3.3)***	22 (1.71)		27.81 (6.42)
WC (cm)		110 (6.24)***	81.8 (6.27)		96.57 (15.54)
Body fat (%)		38.77 (7.91)***	23.25 (4.62)		31.38 (10.18)
DEBQ’S scores					
Emotional		2.88 (0.99)**	1.71 (0.32)		2.26 (1.41)
External		3.31 (0.68)**	1.96 (0.29)		2.83 (0.89)
Restrained		1.81 (0.7)^ns^	1.3 (0.30)		1.75 (0.9)

***P < 0.001, **P < 0.01, *P < 0.05, ns; no significant. Values expressed mean (SD) or % (n)

Regarding the DEBQ results, there were significant differences between individuals with obesity and with normal BMI; the participants with obesity have higher scores on the three eating styles compared to normal BMI group. However, no statistically significant differences were found in the restrained eating subscale.

Using simple linear regression, emotional and external eating were associated with higher BMI (Table 2). However, restraint eating did not show any significant association with BMI.

**Table 2:** Simple linear regression model to predict associations between eating styles and BMI

DEBQ factors	BMI		
	OR	95% CI	P
Emotional	1.216	0.295–2.137*	0.012
External	1.764	0.098–3.429*	0.039
Restraint	0.186	0.434–0.805	0.539

95% CI; Results are expressed as odds ratio (OR) with a 95% confidence interval (95% CI). *P < 0.05

## Discussion

Overeating is an uncontrolled eating behaviour which is closely associated to obesity. Three styles of eating behaviour underlie overeating: emotional, external and restrained eating. In this study, we aimed to assess eating styles of Algerian adult individuals by using the DEBQ. The main objectives were to compare two groups: the first one with normal BMI and WC and normal rate of BF %, the second one with obesity, high WC and high BF%. We also examined the relationship between eating styles and BMI. Findings suggest that there were differences between the two groups on all the eating styles: participants with obesity reported higher score in all DEBQ subscales than normal BMI individuals with tendency for significant high emotional and external eating, supporting previous researches [32, 33]. However, they showed a low restraint score; the difference was no significant versus participants with normal BMI.

Eating in response to emotional factors is acknowledged as a high risk factor for developing overweight, obesity, diabetes and heart disease [33–35]. In response to the negative emotions, participants with obesity and overweight show less effective coping skills leading them to eat more than normal BMI people [36, 37]. It was suggested that 60% or more of individuals with overweight or obesity are emotional eaters [38]. Emotional eaters are less aware of their internal hunger and satiety cues, partially because emotional eating is related to stress which alters awareness to these internal cues [39, 40]. They are likely to consume energy-dense food [14] and exhibit difficulties with weight loss [41]; decreased emotional eating was significantly associated with greater weight loss [42]. However, emotion regulation difficulties may be implicated in weight control following successful weight loss [43, 44]. External eating is a relevant style in obesity that predicts food cravings [45]. Individuals with obesity or overweight were susceptible to environmental cues that contribute to overeating [22]; they include high dietary variety [46], food availability [18] and food palatability [47].

In the modern food environment, the ready availability of high palatable food contributes to hedonic hunger [21]. Study revealed an association between an individual’s hedonic hunger and overeating frequency [48]. It is known that eating responds to hedonic requirement in addition to hunger signals, hedonic proprieties of food influence eating independently to energy status and palatable food can be consumed just for pleasure [49].

In the current study, participants with obesity showed high emotional and external eating styles concomitant to a low restraint, leading to overeat and weight gain. In a previous study including patients with newly diagnosed type 2 diabetes, overeating without restraint was associated with weight gain [50]. Emotional eating tends to co-occur with external eating. Correlation analysis among eating styles and BMI showed positive associations between BMI and both emotional and external eating, as shown by previous researches [32, 51, 52]. The strongest positive association was found for emotional eating. However, a restrained style did not correlate significantly with BMI, which is consistent with other researches [32, 53], and contrary to another that included only individuals with overweight [52].

These variations of restrained associations with BMI change may be explained partly by the fact that restraint is not a one-dimensional construct. In effect, restraint can be rigid (all-or-nothing approach to limiting types and quantities of food) or flexible (approach to limit quantities of certain food) [54]. Rigid and flexible restraint are oppositely associated with obesity and together, they may show no association with BMI [53]. In another part, in response to external or emotional factors, restraint eating can induce disinhibited overeating as counter-regulatory response [55].

There are a few limitations in the current study. The study only included participants with obesity and normal BMI. Future study, including more participants and more categories of BMI cut off as participants with overweight, will shed light on the eating habits in other groups. The invited participants were workers at hospital and two universities which means that these results are not completely representative for adults in Algeria. Nevertheless, the current study provides, for the first-time, worthy data on emotional and external eating and their association with obesity in participants that are workers in hospital or university.

## Conclusion

Obviously, eating styles of participants with obesity has largely different from normal BMI individuals. These eating styles behaviours are characterised by a high level of emotional and external eating. The factor most strongly associated with BMI was emotional eating, it is therefore essential to target the eating styles that underlie overeating for treatment and prevention of obesity. It may be difficult to change pathological eating styles; the challenge is to create long-term lifestyle changes to reduce obesity and associated pathologies. Treatment of external and emotional eating should focus on the sensitivity to food cues and teaching emotions regulation skills to avoid overeating as a response of environmental cues and way to cope with negative emotions.

## Data Availability

The datasets generated and analysed during the current study are not publicly available, as individual privacy could be compromised.

## Abbreviations

BF %: Body fat per cent
DEBQ: Dutch Eating Behaviour Questionnaire
WC: waist circumference

## References

[1] World Health Organization. *Global status report on noncommunicable diseases 2014*:World Health Organization. 10.1161/STROKEAHA.115.008097 25873596

[2] Guh DP, Zhang W, Bansback N, Amarsi Z, Birmingham CL, Anis AH (2009). The incidence of co-morbidities related to obesity and overweight: a systematic review and meta-analysis. BMC Public Health.

[3] Chooi YC, Ding C, Magkos F (2019). The epidemiology of obesity. Metab.

[4] Mammeri A, Tebaibia A (2020). Cardiometabolic risk in Algeria: past and present. Intern Emerg Med.

[5] Renner B, Sproesser G, Strohbach S, Schupp HT (2012). Why we eat what we eat. The eating motivation survey (TEMS). Appetite.

[6] Micha R, Peñalvo JL, Cudhea F, Imamura F, Rehm CD, Mozaffarian D (2017). Association between dietary factors and mortality from heart disease, stroke, and type 2 diabetes in the United States. JAMA.

[7] Lennerz BS, Alsop DC, Holsen LM, Stern E, Rojas R, Ebbeling CB, Goldstein JM, Ludwig DS (2013). Effects of dietary glycemic index on brain regions related to reward and craving in men. Am J Clin Nutr.

[8] Gearhardt AN, Grilo CM, Di Leone RJ, Brownell KD (2011). Potenza M. N. can food be addictive? Public health and policy implications. Addiction.

[9] Benbaibeche H, Haffaf EM, Kacimi G, Oudjit B, Khan NA, Koceïr EA (2015). Implication of corticotropic hormone axis in eating behaviour pattern in obese and type 2 diabetic participants. Br J Nutr.

[10] Ouhaibi-Djellouli H, Houti L, Hamani-Medjaoui I, Larjam-Hetraf AS, Mediene-Benchekor S (2020). Mediterranean diet and food consumption in an urban adult population of Northwest Algeria. North Afr J Food Nutr Res.

[11] Huo R, Du T, Xu Y, Xu W, Chen X, Sun K, Yu X (2015). Effects of Mediterranean-style diet on glycemic control, weight loss and cardiovascular risk factors among type 2 diabetes individuals: a meta-analysis. Eur J Clin Nutr.

[12] Van Strien T, Frijters JE, Bergers GP, Defares PB (1986). The dutch eating Behavior Questionnaire (DEBQ) for assessment of restrained, emotional, and external eating behavior. Int J Eat Disord.

[13] Camilleri GM, Méjean C, Kesse-Guyot E, Andreeva VA, Bellisle F, Hercberg S, Péneau (2014). The associations between emotional eating and consumption of energy-dense snack foods are modified by sex and depressive symptomatology. J Nutr.

[14] Elfhag K, Tholin S, Rasmussen F (2008). Consumption of fruit, vegetables, sweets and soft drinks are associated with psychological dimensions of eating behaviour in parents and their 12-year-old children. Public Health Nutr.

[15] Klump KL, O’Connor SM, Hildebrandt BA, Keel PK, Neale M, Sisk CL, Boker S, Burt SA (2016). Differential effects of estrogen and progesterone on genetic and environmental risk for emotional eating in women. Clin Psychol Sci.

[16] Dohle S, Hartmann C, Keller C (2014). Physical activity as a moderator of the association between emotional eating and BMI: evidence from the swiss food panel. Psychol health.

[17] Van Strien T, Cebolla A, Etchemendy E, Gutierrez-Maldonado J, Ferrer-Garcia M, Botella C, Baños R (2013). Emotional eating and food intake after sadness and joy. Appetite.

[18] Elliston KG, Ferguson SG, Schüz N, Schüz B (2017). Situational cues and momentary food environment predict everyday eating behavior in adults with overweight and obesity. Health Psychol.

[19] Burton P, Smit HJ, Lightowler HJ (2007). The influence of restrained and external eating patterns on overeating. Appetite.

[20] McCrory MA, Burke A, Roberts SB (2012). Dietary (sensory) variety and energy balance. Physiol Behav.

[21] Lowe MR, Butryn ML (2007). Hedonic hunger: a new dimension of appetite?. Physiol Behav.

[22] Herman CP, Polivy J (2008). External cues in the control of food intake in humans: the sensory-normative distinction. Physiol Behav.

[23] Allen Kl, Byrne SM, La Puma M, McLean N, Davis E (2008). The onset and course of binge eating in 8- to 13-year-old healthy weight, overweight and obese children. Eat Behav.

[24] Van De Laar FA, Van De Lisdonk EH, Lucassen PL, Stafleu A, Mulder J, Van den Hoogen H, Rutten GEHM, Van Weel C (2006). Eating behaviour and adherence to diet in patients with type 2 diabetes mellitus. Diabet med.

[25] Westenhoefer J, Von Falck B, Stellfeldt A, Fintelmann S (2004). Behavioural correlates of successful weight reduction over 3 y. results from the lean Habits Study. Int J Obes.

[26] Deurenberg P, Weststrate JA, Seidell JC (1991). Body mass index as a measure of body fatness: age-and sex-specific prediction formulas. Br J Nutr.

[27] Khanna D, Rehman A (2022). Pathophysiology of obesity.

[28] Buss J (2014). Limitations of body mass index to assess body fat. Workplace Health Saf.

[29] Flegal KM, Shepherd JA, Looker AC, Graubard BI, Borrud LG, Ogden CL, Harris TB, Everhart JE, Schenker N (2009). Comparisons of percentage body fat, body mass index, waist circumference, and waist-stature ratio in adults. Am J Clin Nutr.

[30] Aeberli I, Gut-Knabenhans M, Kusche-Ammann RS, Molinari L, Zimmermann MB (2013). A composite score combining waist circumference and body mass index more accurately predicts body fat percentage in 6- to 13-year-old children. Eur J Nutr.

[31] Galmiche M, Déchelotte P, Lambert G, Tavolacci MP (2019). Prevalence of eating disorders over the 2000–2018 period: a systematic literature review. Am J Clin Nutr.

[32] Baños RM, Cebolla A, Moragrega I, Van Strien T, Fernández-Aranda F, Agüera Z, De La Tore F, Casnueva FF, Fernandez-Real JM, Fernandez-Garcia JC, Frühbeck G, Gomez-Ambrosi J, Jiménez-Murcia S, Rodriguez R, Tinahones FJ, Botella C (2014). Relationship between eating styles and temperament in an Anorexia Nervosa, Healthy Control, and morbid obesity female sample. Appetite.

[33] Van Strien T, Herman CP, Verheijden MW (2012). Eating style, overeating and weight gain. A prospective 2-year follow-up study in a representative dutch sample. Appetite.

[34] Koenders PG, Van Strien T. Emotional eating, rather than lifestyle behavior, drives weight gain in a prospective study in 1562 employees. J Occup Environ Med 2011: 1287–93. 10.1097/JOM.0b013e31823078a2 22027541

[35] Wang H, Shara NM, Calhoun D, Umans JG, Lee ET, Howard BV (2010). Incidence rates and predictors of diabetes in those with prediabetes: the strong heart study. Dib/Metab Res Rev.

[36] Bourdier L, Fatseas M, Maria A-S, Carre A, Berthoz S (2020). The psycho-affective roots of obesity: results from a french study in the general population. Nutr.

[37] Ozier AD, Kendrick OW, Leeper JD, Knol LL, Perko M, Burnham J (2008). Overweight and obesity are associated with emotion-and stress-related eating as measured by the eating and appraisal due to emotions and stress questionnaire. J Am Diet Assoc.

[38] Ganley RM (1989). Emotion and eating in obesity: a review of the literature. Intl J Eat Disord.

[39] Tan CC, Chow CM (2014). Stress and emotional eating: the mediating role of eating dysregulation. Personal Indiv differ.

[40] Goossens L, Braet C, Decaluwé V (2007). Loss of control over eating in obese youngsters. Behav Res Ther.

[41] Butryn ML, Thomas JG, Lowe MR (2009). Reductions in internal disinhibition during weight loss predict better weight loss maintenance. Obes.

[42] Braden A, Flatt SW, Boutelle KN, Strong D, Sherwood NE, Rock CL (2016). Emotional eating is associated with weight loss success among adults enrolled in a weight loss program. J Behav Med.

[43] Feller S, Müller A, Mayr A, Engeli S, Hilbert A, De Zwaan M (2015). What distinguishes weight loss maintainers of the G erman W eight C ontrol R egistry from the general population?. Obes.

[44] Geliebter A, Aversa A (2003). Emotional eating in overweight, normal weight, and underweight individuals. Eat Behav.

[45] Moreno-Domínguez S, Rodríguez‐Ruiz S, Fernández‐Santaella MC, Ortega‐Roldán B, Cepeda‐Benito A (2012). Impact of fasting on food craving, mood and consumption in bulimia nervosa and healthy women participants. Eur Eat Disord Rev.

[46] Bounihi A, Saidi H, Bouazza A, Benbaibeche H, Azzouz M, Koceir EA (2021). Dietary diversity as a risk factor for obesity in algerian patients with type 2 diabetes Mellitus. Healthc.

[47] Monteiro CA (2009). Nutrition and health. The issue is not food, nor nutrients, so much as processing. Public Health Nutr.

[48] Horwath CC, Hagmann D, Hartmann C (2020). The power of food: self-control moderates the association of hedonic hunger with overeating, snacking frequency and palatable food intake. Eat Behav.

[49] Shomaker LB, Tanofsky-Kraff M, Zocca JM, Courville A, Kozlosky M, Columbo KM, Wolkoff LE, Brady SM, Crocker MK, Ali AH, Yanovski SZ, Yanovski JA (2010). Eating in the absence of hunger in adolescents: intake after a large-array meal compared with that after a standardized meal. Am J Clin Nutr.

[50] Van Strien T, Van De Laar FA, Van Leeuwe JF, Lucassen PL, Van Den Hoogen HJ, Rutten GE, Van Weel C (2007). The dieting dilemma in patients with newly diagnosed type 2 diabetes: does dietary restraint predict weight gain 4 years after diagnosis?. Health Psychol.

[51] Konttinen H, Haukkala A, Sarlio-Lähteenkorva S, Silventoinen K, Jousilahti P (2009). Eating styles, self-control and obesity indicators. The moderating role of obesity status and dieting history on restrained eating. Appetite.

[52] Van Strien T, Herman CP, Verheijden MW (2009). Eating style, overeating, and overweight in a representative dutch sample. Does external eating play a role?. Appetite.

[53] Provencher V, Drapeau V, Tremblay A, Després JP, Lemieux S (2003). Eating behaviors and indexes of body composition in men and women from the Quebec family study. Obes Res.

[54] Westenhoefer J, Engel D, Holst C, Lorenz J, Peacock M, Stubbs J, Whybrow S, Raats M (2013). Cognitive and weight-related correlates of flexible and rigid restrained eating behaviour. Eat Behav.

[55] Johnson F, Pratt M, Wardle J (2012). Dietary restraint and self-regulation in eating behavior. Int J Obes.

